# A Malignant Connection: Bronchoesophageal-Pleural Fistula in an Elderly Farmer

**DOI:** 10.7759/cureus.27966

**Published:** 2022-08-13

**Authors:** Sam Gaine, Hammad Danish, Wail Binalialsharabi, Sean Fennessy, Ashraf Morcos, Mark Rogan

**Affiliations:** 1 Department of Rheumatology, University Hospital Waterford, Waterford, IRL; 2 Department of General Internal Medicine, University Hospital Waterford, Waterford, IRL; 3 Department of Pulmonology, University Hospital Waterford, Waterford, IRL; 4 Department of Gastroenterology, University Hospital Waterford, Waterford, IRL

**Keywords:** bronchoesophageal-pleural fistula, broncho-esophageal fistula, self-expanding metal stents, oesophageal stents, esophageal squamous cell carcinoma (scc), oesophageal cancer, oesophagobronchial, brochoesophageal

## Abstract

Bronchoesophageal-pleural fistula (BEPF) is a very rare entity that can present as a late manifestation of oesophageal malignancy. Here, we describe the case of an elderly farmer with no past medical history of note who presented with acute respiratory failure associated with a five-month history of dysphagia and weight loss.

Computerised tomography of the thorax showed a connection between the oesophagus, bronchus and pleural space: a bronchoesophageal-pleural fistula. Ultrasound-guided thoracentesis was followed by chest drain insertion into an empyema containing food debris. Histopathological analysis of endoscopic biopsies confirmed an eroding squamous cell carcinoma (SCC) of the oesophagus. An oesophageal stent was inserted to seal off the fistula and broad-spectrum antibiotics were maximised. Ultimately, after four weeks in hospital, palliative therapy was initiated.

BEPF remains a very rare and devastating complication of oesophageal malignancy. Endoscopic stenting may provide symptomatic relief.

## Introduction

A bronchoesophageal-pleural fistula (BEPF) is an abnormal tract between the oesophagus, a major bronchus, and the pleural space [1]. Very few case reports have been published describing such a connection. Bronchoesophageal fistulae arise as a complication of oesophageal malignancy in 50% of cases. Rarely, they can also occur due to other malignancies (e.g., bronchogenic), infections (e.g., TB), or iatrogenic causes (e.g., prolonged intubation, radiotherapy) [2]. Here, we report the case of an elderly man who presented with BEPF secondary to an eroding squamous cell carcinoma (SCC) of the oesophagus, leading to the development of an empyema containing food debris.

## Case presentation

A 71-year-old single farmer with no past medical history presented to the emergency department due to acute respiratory failure.

Five months previously, he noticed dysphagia associated with a reduced appetite and loss of weight. He also experienced bouts of coughing after eating. He was an ex-smoker with a 20-year past history. He had no prior hospitalisations.

On examination, the patient was febrile (38.5 C). He was markedly cachectic and in acute respiratory distress. Coarse crackles were noted in his right middle and lower zones bilaterally. Blood gases showed a Type 1 respiratory failure. Other laboratory results were significant for acute kidney infection (AKI), hyponatremia, hypoalbuminemia, raised C-reactive protein (CRP), and leucocytosis with a neutrophilic predominance. He was commenced on high-flow oxygen, intravenous IV antibiotics and IV fluids.

**Figure 1:**
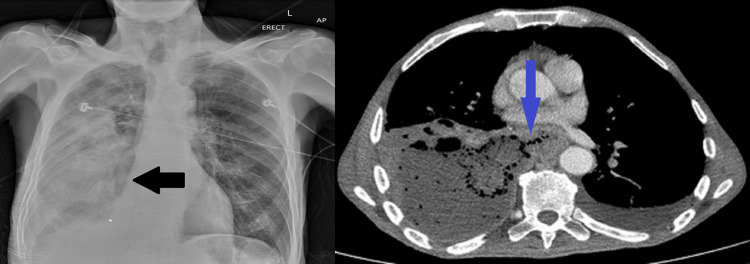
Chest X-ray: right-sided mid-lower zone infiltrates (black arrow); CT thorax: Diffuse oesophageal thickening with right-sided necrotising pneumonia, 14 cm abscess and bronchoesophageal-pleural fistula (BEPF) (blue arrow)

Chest X-ray (Figure 1, black arrow) showed infiltrates in the right mid-lower zones and a small pleural effusion. CT thorax (Figure 1, blue arrow) revealed oesophageal mural thickening, necrotising pneumonia, a 14-cm right-sided abscess, and a connection between the oesophagus and right main bronchus with pleural involvement: a bronchoesophageal-pleural fistula.

The patient was kept ‘nil by mouth’ and started on total parenteral nutrition. He underwent ultrasound-guided thoracentesis followed by chest drain insertion into an empyema, which consisted of frank pus, an abundance of neutrophils, candida and enterococcus species, and, interestingly, food debris. Microbiological analysis of pleural fluid demonstrated the presence of acid-fast bacilli, which were subsequently identified as non-tuberculous mycobacteria.

**Figure 2:**
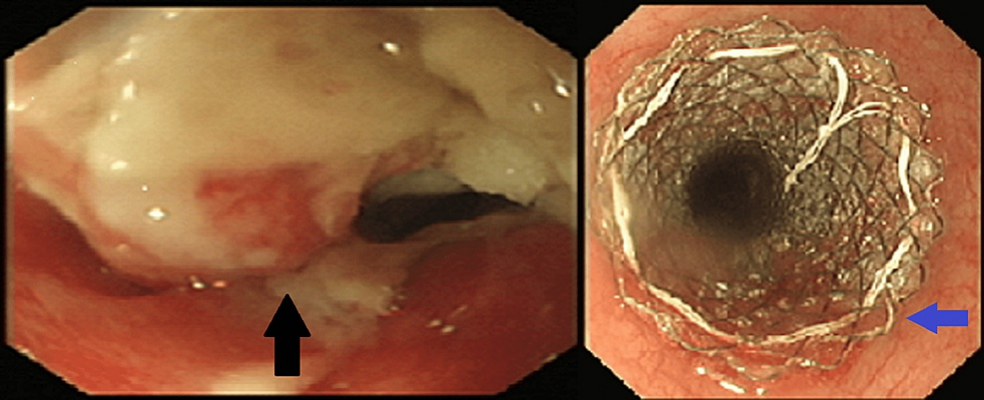
OGD: Narrowing and necrotic mass at the mid-to-lower oesophagus (black arrow), treated with a covered, self-expanding metal stent (blue arrow) OGD: oesophagogastroduodenoscopy

Oesophagogastroduodenoscopy (OGD) was performed to further characterise the lesion (Figure 2, black arrow), which was thickened circumferentially but allowed free passage of the endoscope distally. A covered, self-expanding metal stent (Figure 2, blue arrow) was inserted to allow oral feeding and improve symptom control. Histopathological analysis of biopsy samples confirmed SCC of the oesophagus.

Palliative measures were commenced, and the patient passed away four weeks after the initial presentation.

## Discussion

A bronchoesophageal-pleural fistula (BEPF) is a very rare entity compared to the more prevalent bronchoesophageal fistula, which complicates as many as 10% of oesophageal cancer cases. About half of bronchoesophageal fistulas are due to malignancy, occasionally bronchogenic but usually oesophageal [1-2].

A non-malignant bronchoesophageal fistula in the adult is rare. Benign aetiologies include infections (e.g., TB), iatrogenic causes (e.g., prolonged intubation, radiotherapy) and congenital anomalies [3].

Nonspecific symptoms can lead to a delay in diagnosis. Such delays may be complicated by pneumonia, life-threatening haemoptysis and respiratory failure. Oesophagography provides a definitive diagnosis in 78% of cases, and OGD with biopsy is performed if imaging is suggestive [3].

The clinical features of malignant BEPF include cough (56%), aspiration (37%) and dysphagia (19%) [4]. Bouts of coughing while eating or drinking (Ohno's sign) and recurrent pneumonia are common late signs. The most common respiratory location for an abnormal tract includes the trachea (53%), followed by the left main bronchus (22%); only 16% affect the right main bronchus, as in this case [4]. Oesophageal contact with pleura occurs for a considerable distance on the right side. Consequently, there is a greater chance of right pleural involvement, consistent with our patient’s presentation [5].

BEPF is a devastating complication of advanced oesophageal SCC [1]. In addition to oesophageal bypass, surgical intervention can be a viable option in select patients and chemoradiation therapy may confer a survival benefit compared with supportive care alone [6]; however, our patient was neither a candidate for radical systemic therapy nor surgical bypass. One study reported symptomatic relief in 90% of patients treated by SEM stent insertion, and a similar rate of success in the closure of these malignant fistulae [7].

As a late manifestation of the disease, BEPF further limits the prognosis in these patients. A recent study found that patients with oesophageal fistula in advanced oesophageal SCC had a median overall survival time of 11 months and a median post-fistula survival time of 3.6 months [8].

In conclusion, BEPF is a very rare entity associated with significant morbidity and mortality. The unusual finding of food debris in this patient's pleural fluid helped differentiate the underlying pathological connection from the more prevalent bronchoesophageal fistula.

## Conclusions

BEPF is a very rare and devastating complication of oesophageal malignancy. As an additional sign, the clinical finding of food debris in the pleural aspirate may guide the clinician in ordering further investigations and identifying the underlying pathological connection. Endoscopic stenting may provide symptomatic relief in these terminal cases.
